# Worldwide Regional Differences in Obesity, Elderly, and COVID-19 Mortality: Do the Exceptions Prove the Rule?

**DOI:** 10.18103/mra.v11i8.4248

**Published:** 2023-08-29

**Authors:** James A. Koziol, Jan E. Schnitzer

**Affiliations:** Proteogenomics Research Institute for Systems Medicine La Jolla, California USA

## Abstract

**Objectives.:**

Obesity and old age are commonly assumed to be risk factors for COVID-19 mortality. On a worldwide basis, we examine quantitative measures of obesity and elderly in the populations of individual countries and territories, and investigate whether these measures are predictive of COVID-19 mortality in those countries. In particular, we highlight regional differences relative to obesity and elderly metrics, and how these relate to COVID-19 mortality.

**Methods.:**

In this retrospective, population-based study, we obtained data relating to percentages of obese or elderly individuals in 199 countries, as well as COVID-19 mortality rates in these countries. We used negative binomial regression analyses to assess associations between COVID-19 mortality rates and the putative risk factors, in six regions – Africa, Asia, Europe, North America, Oceania, and South America.

**Results.:**

We found significant differences between regions relative to COVID-19 mortality, as well as obesity and elderly population proportions. There were also substantial differences between countries within regions relative to proportions of obesity and elderly individuals, and COVID-19 mortality.

**Conclusions.:**

There are significant differences both between regions and within regions relative to COVID-19 mortality rates, as well as proportions of obese or elderly individuals. A global pronouncement that obesity and elderly constitute definitive risk factors for COVID-19 mortality masks the subtleties engendered by these intra- and inter-regional differences.

## Introduction

The COVID-19 pandemic is a global health crisis, resulting in significant morbidity and mortality which has affected millions of people worldwide. The virus disproportionately affects certain populations, in particular, the elderly and those with preexisting health conditions such as obesity. It is commonly accepted that obesity and elderly age are significant risk factors for severe illness and death from COVID-19. Obesity can impair lung function, increasing the risk of respiratory infections such as COVID-19, and is associated with chronic low-grade inflammation, which can weaken the immune system and make it harder to fight off infections. It is often associated with a range of comorbidities, including diabetes, hypertension, and cardiovascular disease, all of which can further increase the risk of severe illness from COVID-19. Similarly, aging is associated with a weakening of the immune system, and elderly individuals are more likely to have underlying health conditions including obesity and associated comorbidites, again increasing the risk of severe illness or mortality from COVID-19.

Relationships between obesity, elderly population, and COVID-19 morbidity and mortality have been extensively investigated^1–16^ ; but here we take a more nuanced approach. On a worldwide basis, we examine quantitative measures of obesity and elderly in the populations of individual countries and territories, and investigate whether these measures are predictive of COVID-19 mortality in those countries. Unlike previous publications, we do not adopt a meta-analytic perspective, as we recognize that there is extensive heterogeneity across countries and regions relative to obesity and elderly population profiles, and how these relate to COVID-19 mortality. We believe this heterogeneity presents challenges to the orthodoxy that obesity and old age are universal risk factors for COVID-19 mortality.

We highlight these regional differences in our synthesis of the obesity and elderly data, and discuss implications of these findings relative to observed COVID-19 mortality.

## Methods

All data used in this study are freely available to the public, in a totally anonymized format without any identifiable information on a personal level. This study was therefore considered exempt from ethical review by our institution.

### Data

Cumulative totals of mortality attributable to COVID-19 per country or territory were obtained from the World Health Organization’s coronavirus dashboard ^17^. Elderly population percentages, with elderly constituting individuals aged 65 and above, were obtained from the World Bank DataBank ^18^ , as determined with data from the United Nations Population Division. We used percentages from 2021 in our study. Obesity population percentages, with obesity defined as body mass index of 30 or greater, were obtained from the Bariatrics Report ^19^, 2022 data.

We merged the three datasets, with country or territory as key. This resulted in a combined dataset representing 199 countries or territories. For some analyses and graphs, we subdivided these data by region: Africa, Asia, Europe, North America, Oceania, or South America.

### Statistical Methods

Our study is largely observational, hence we are hesitant to overly analyze these data. Nevertheless, we do utilize regression methodology to tease out any relations between population proportions of elderly or obesity and region, and associations between COVID-19 mortality and regional obesity and elderly proportions. Our main tool in this regard is negative binomial regression^20–22^. We remark in passing that Poisson regression models are commonly used for modeling count data, but we initially found that obesity and elderly proportions as well as COVID-19 deaths across countries are substantially over-dispersed relative to the Poisson assumption that mean is equal to the variance. Thus a Poisson distributional assumption for our endpoints of interest appeared inappropriate, and we chose to adopt negative binomial models, that allow count outcome variables with variance larger than the mean.

Formally, let Y denote a (dependent) variable that is an observed count following a negative binomial distribution. We may write

$$\mathrm{Pr}\left(Y=y_{i}|\mu_{i},\alpha \right)=\frac{\text{Γ}\left(y_{i}+\alpha^{-1}\right)}{\text{Γ}\left(y_{i}+1\right)\text{Γ}\left(\alpha^{-1}\right)}{\left(\frac{\alpha^{-1}}{\alpha^{-1}+\mu_{i}}\right)}^{\alpha^{-1}}{\left(\frac{\mu_{i}}{\alpha^{-1}+\mu_{i}}\right)}^{y_{i}}$$

where $\mu_{1}={\text{p}}_{\text{i}}\mu$ and α is sometimes parameterized by α=1/v. The parameter μ is the mean incidence rate of Y per unit of exposure. In our setting, exposure constitutes population size, and we use the symbol p_i_ to represent the underlying population size (the population at risk) for a particular observation.

Turning to the negative binomial regression model, the mean of Y is determined by the population size p and a set of kregressor variables (conventionally, the x’s in a linear regression Y=AX+b). The expression relating these entities is

$$\mu_{i}=Exp\left(Log\left(p_{i}\right)+\sum_{j=1}^{k}\beta_{j}x_{ji}\right).$$

Here, Exp() is the exponential function, and Log() denotes the natural log. Often, we set ${\text{x}}_{1}=1$, and ${\text{β}}_{1}$ is termed the intercept. The regression coefficients, the ${\text{β}}_{\text{j}}$, are unknown parameters that are then estimated from a set of data.

We utilized negative binomial regressions to compare obesity, elderly, and COVID-19 mortality between regions: the ${\text{Y}}_{\text{i}}$ are counts of obesity, elderly, or mortality per country or territory, the p_i_ are the corresponding populations, and the ${\text{x}}_{\text{i}}$ are indicator variables for the 6 regions of interest.

Within each region, we also utilized negative binomial regressions to examine associations between COVID-19 mortality and obesity or elderly. In this scenario, the ${\text{Y}}_{\text{i}}$ are counts of mortality for country i, the p_i_ are corresponding population sizes, k=2, the ${\text{x}}_{1\text{i}}$ are all 1 and the ${\text{x}}_{2\text{i}}$ are percentages of obesity or elderly per country. Hence for each region we are estimating an intercept and slope of a regression line relating obesity or elderly to COVID-19 mortality for the countries in that region, a regression line that is linear in log (mortality rate).

## Results

**Figure 1.**
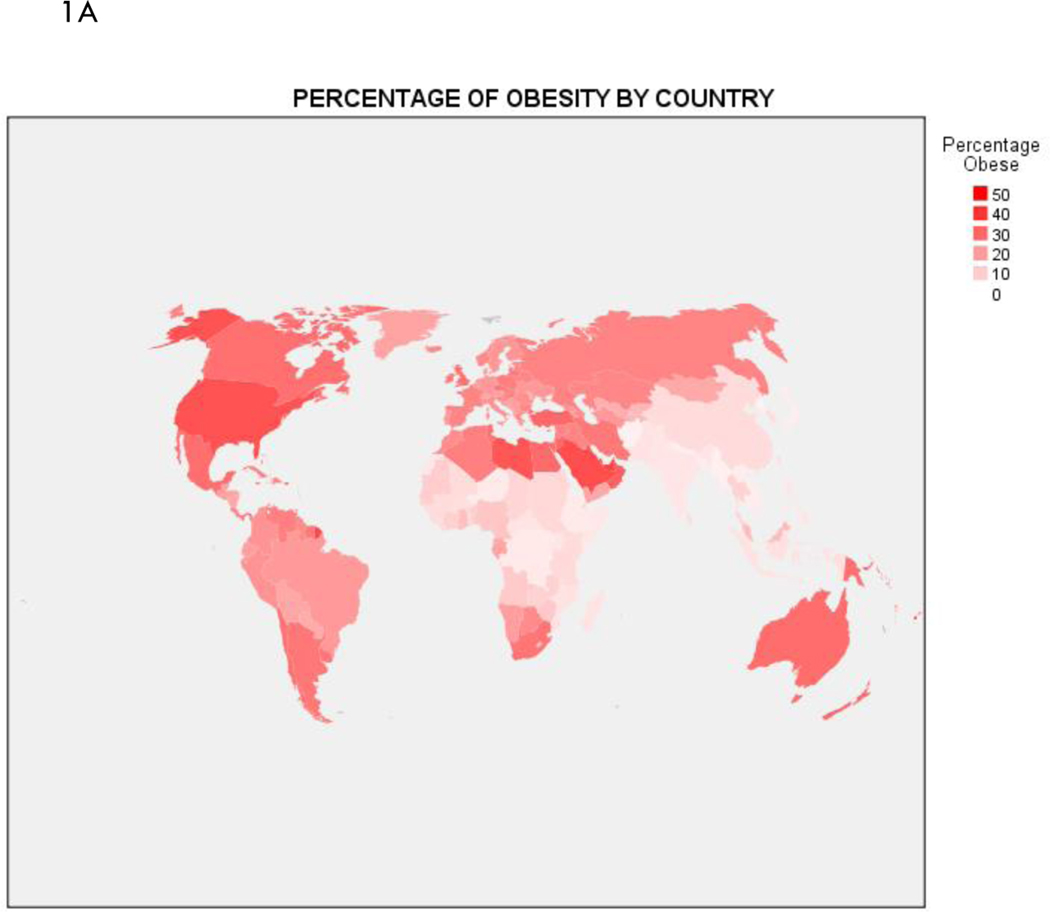

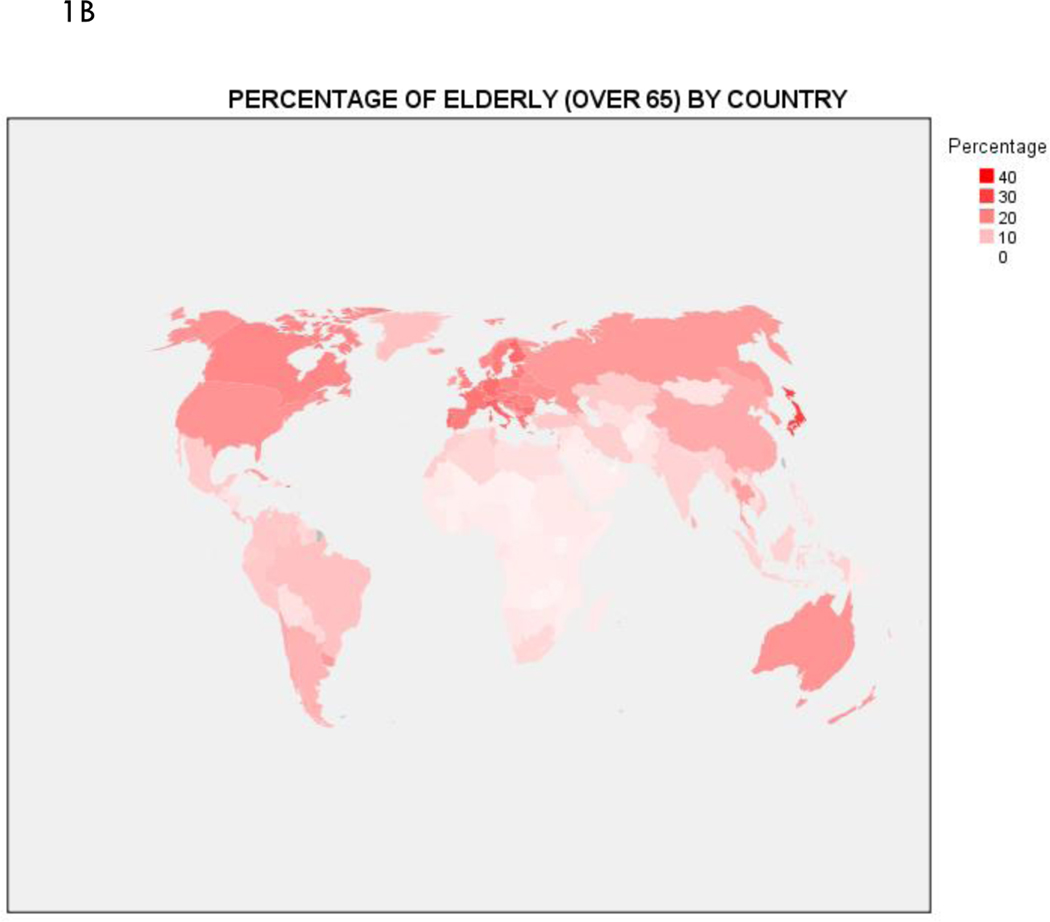
World choropleths of population percentages of (A) obesity and (B) elderly by country. Obesity represents body mass index of 30 or greater, and elderly constitutes individuals aged 65 or older.

**Figure 2.**
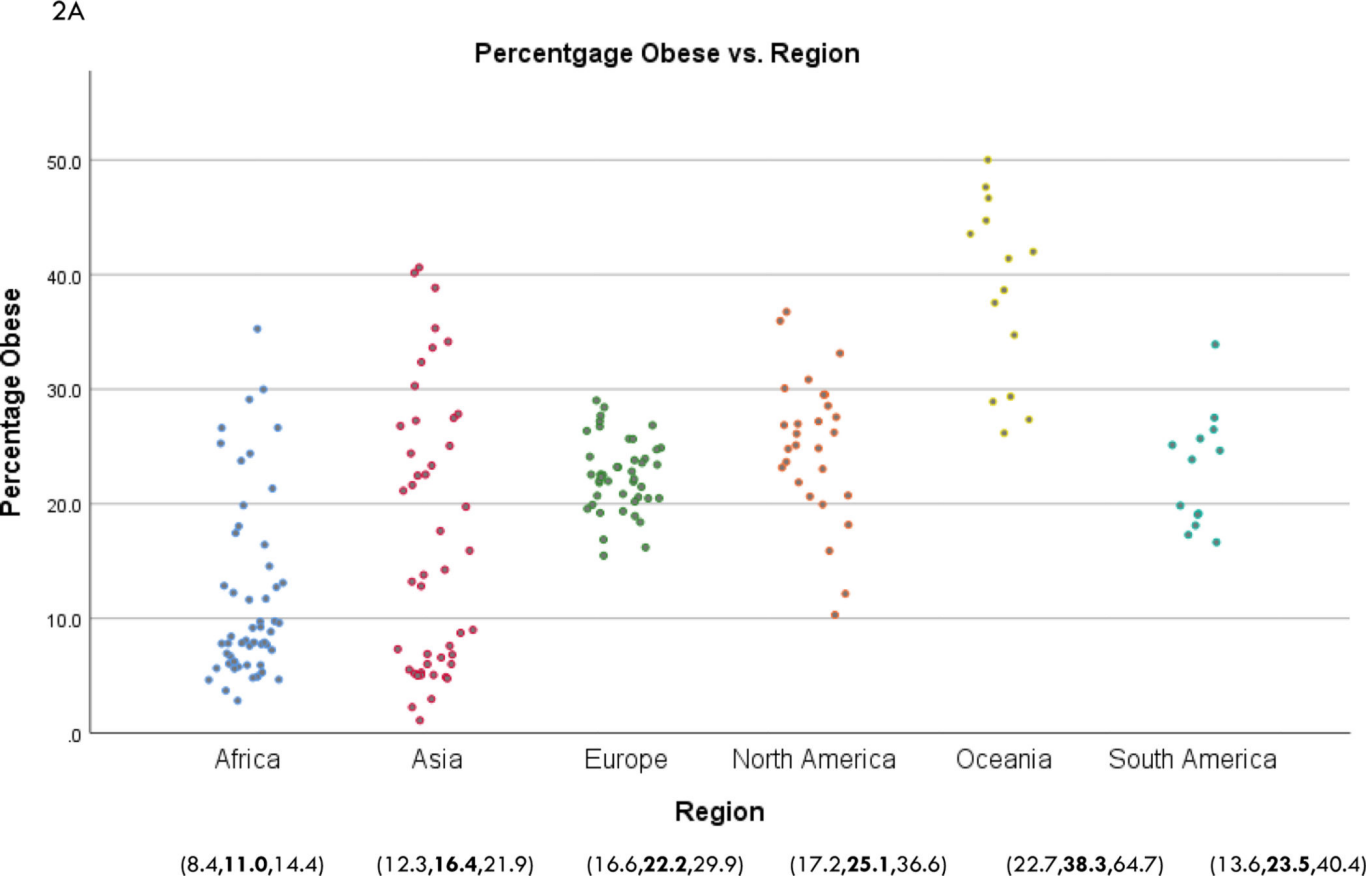

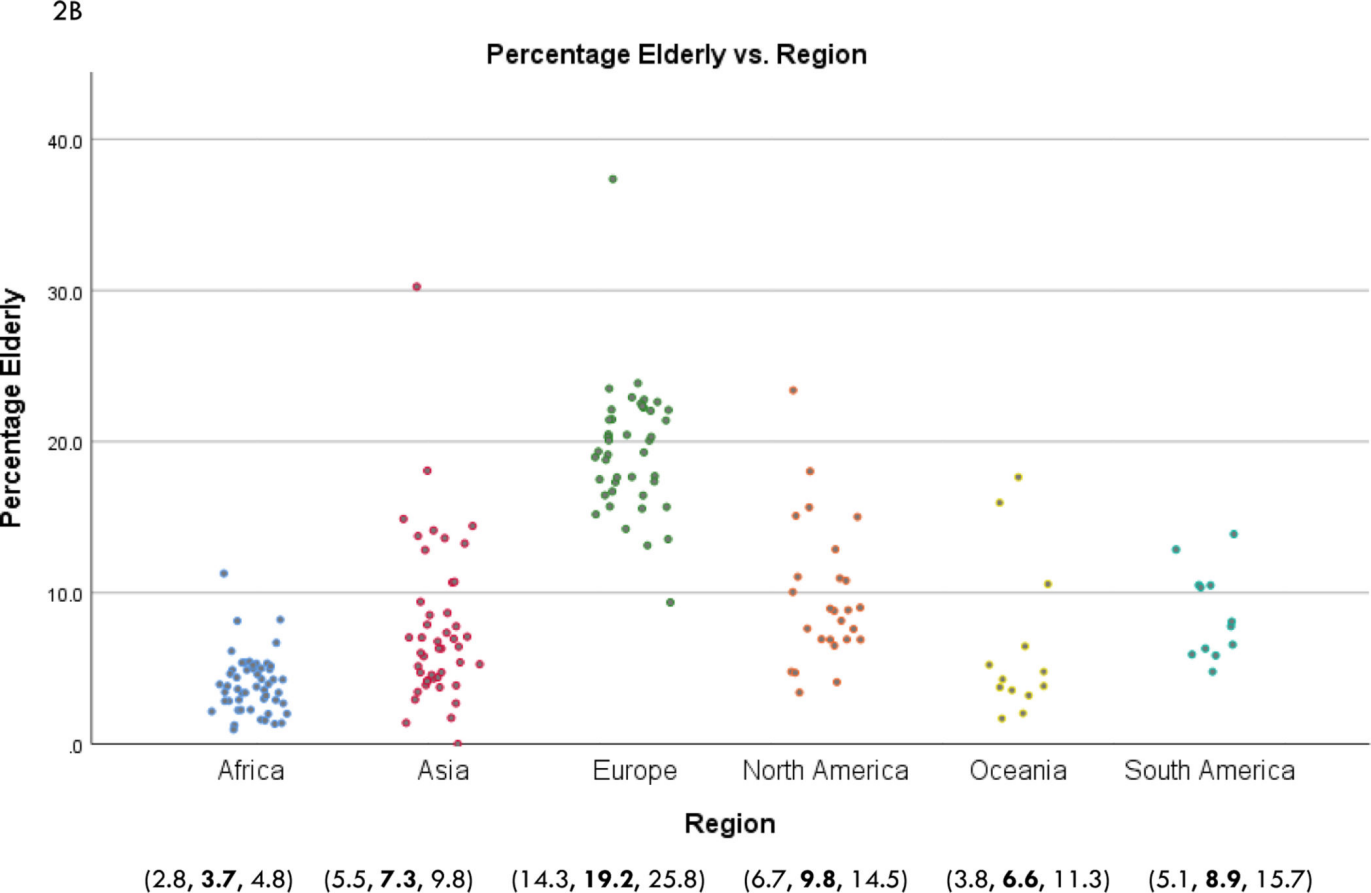
Percentages of (A) obesity and (B) elderly by region. Countries, from Figure 1, were classified into 6 regions: Africa, Asia, Europe, North America, Oceania, and South America. The numbers underneath the regions are the estimated marginal means of the respective percentages (middle numbers) along with associated 95% confidence limits (flanking numbers). These were obtained from negative binomial regressions per region, as explained in the text.

In Figure 1 we present global choropleths of percentages of obesity and elderly. Relatively high levels of obesity seem common throughout the world, with the exceptions of countries in eastern Asia and central Africa. The elderly profile is quite different: with the exception of the Africa region, countries tend to have a substantial proportion of elderly people. A slightly different perspective of national differences in obesity and elderly is given in Figure 2, where we give scatter diagrams of these percentages by region, namely, Africa, Asia, Europe, North America, Oceania, and South America. We also give in Figure 2 estimated marginal means of regional percentages of obesity and elderly from negative binomial regressions, along with associated 95% confidence intervals. Notably, compared to other regions, Africa overall exhibits significantly less obesity and elderly, and, Europe has a significantly higher percentage of elderly. In each region, there is less variability in elderly compared to obesity proportions.

**Figure 3.**
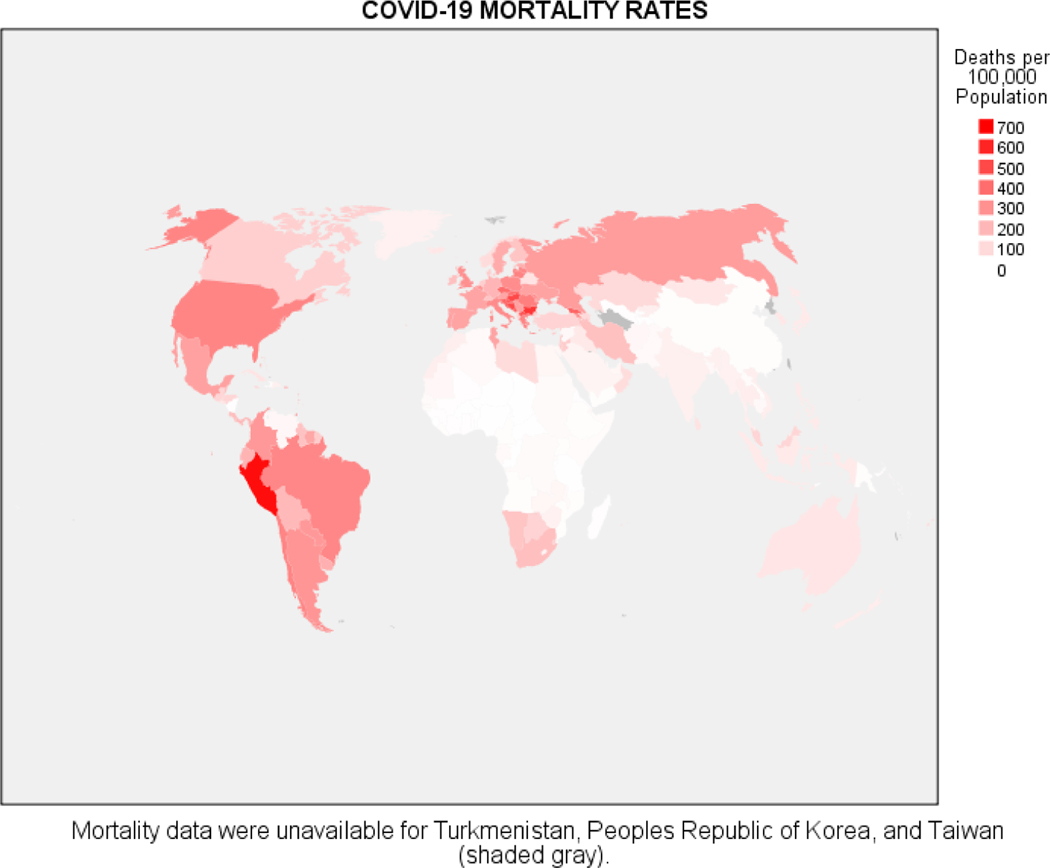
World choropleth of COVID-19 mortality rates by country.

**Figure 4.**
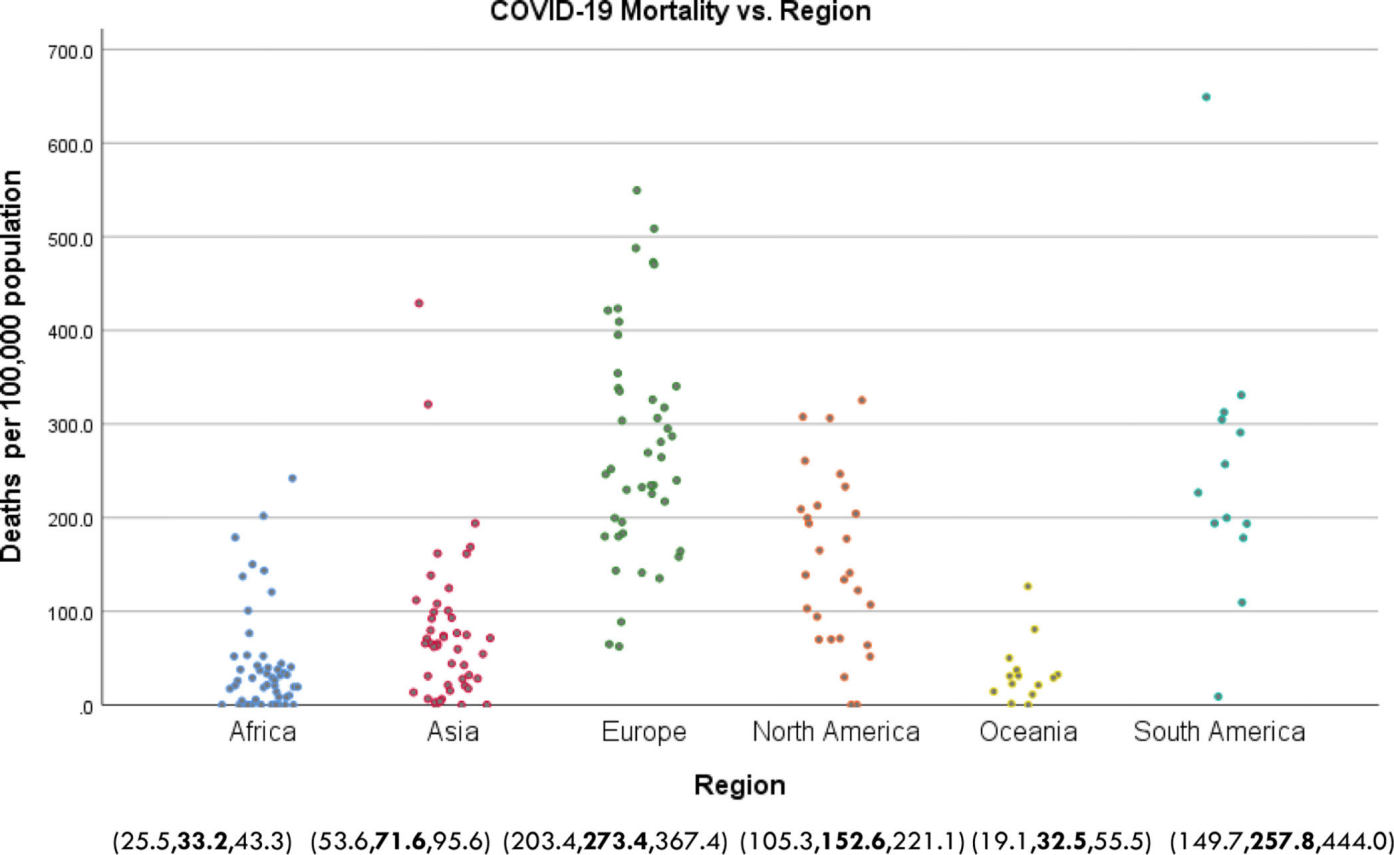
COVID-19 mortality rates by region (as in Figure 2), along with estimated marginal means and associated 95% confidence limits.

We next turn to COVID-19 mortality, beginning with a global choropleth in Figure 3. Differences between regions are perhaps more pronounced than found in Figure 1, with Africa in particular faring quite well on this metric. Figure 4 is analogous to Figure 2, a scatter diagram of COVID-19 mortality by region, along with estimated marginal means of mortality and associated 95% confidence intervals. The regions comprise two relatively homogeneous subgroups: Africa, Asia, and Oceania experienced significantly less COVID-19 mortality than Europe, North America, and South America. Oceania is relatively homogeneous relative to COVID-19 mortality, but again there is substantial variability in mortality rates in the other regions.

**Figure 5.**
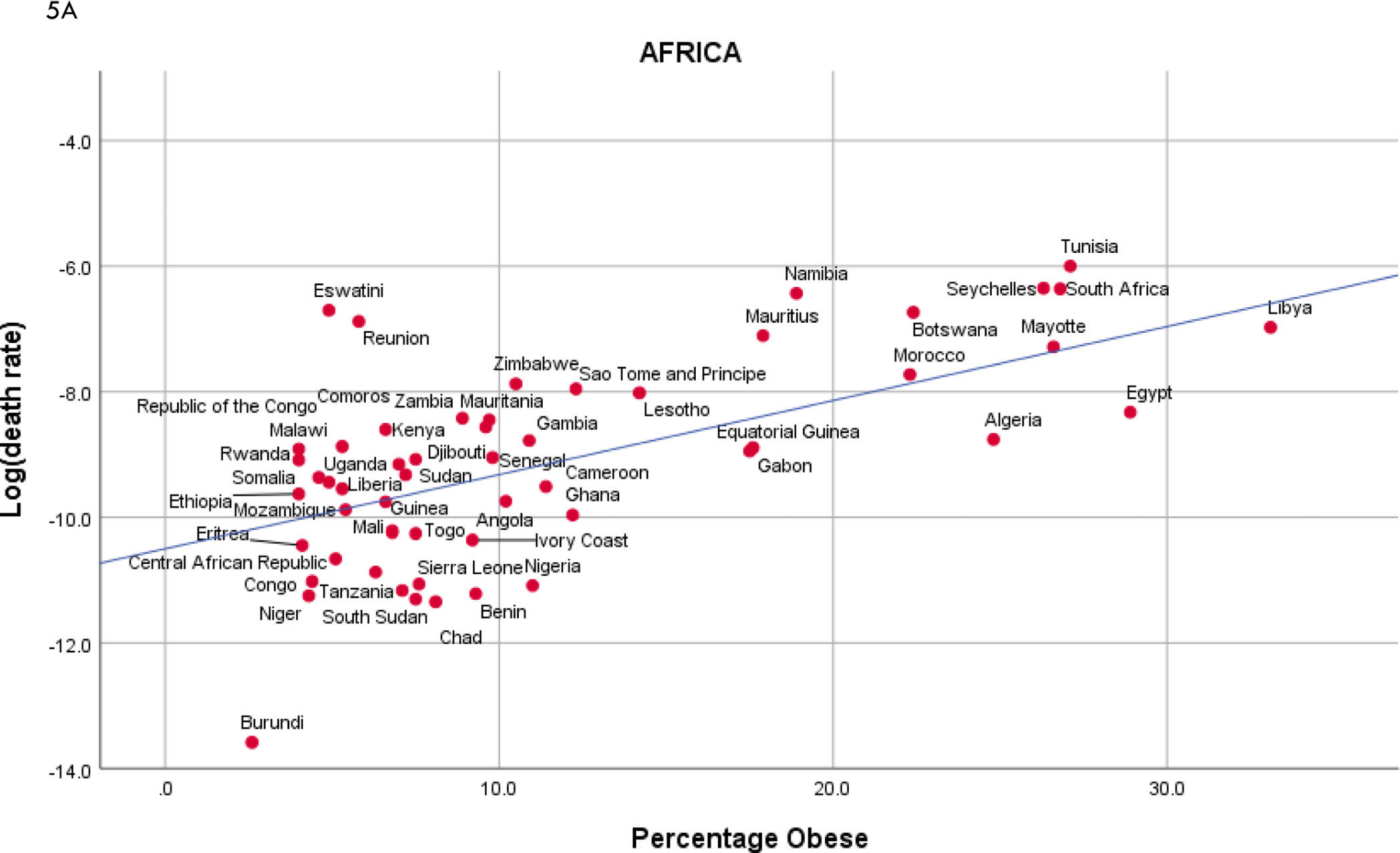

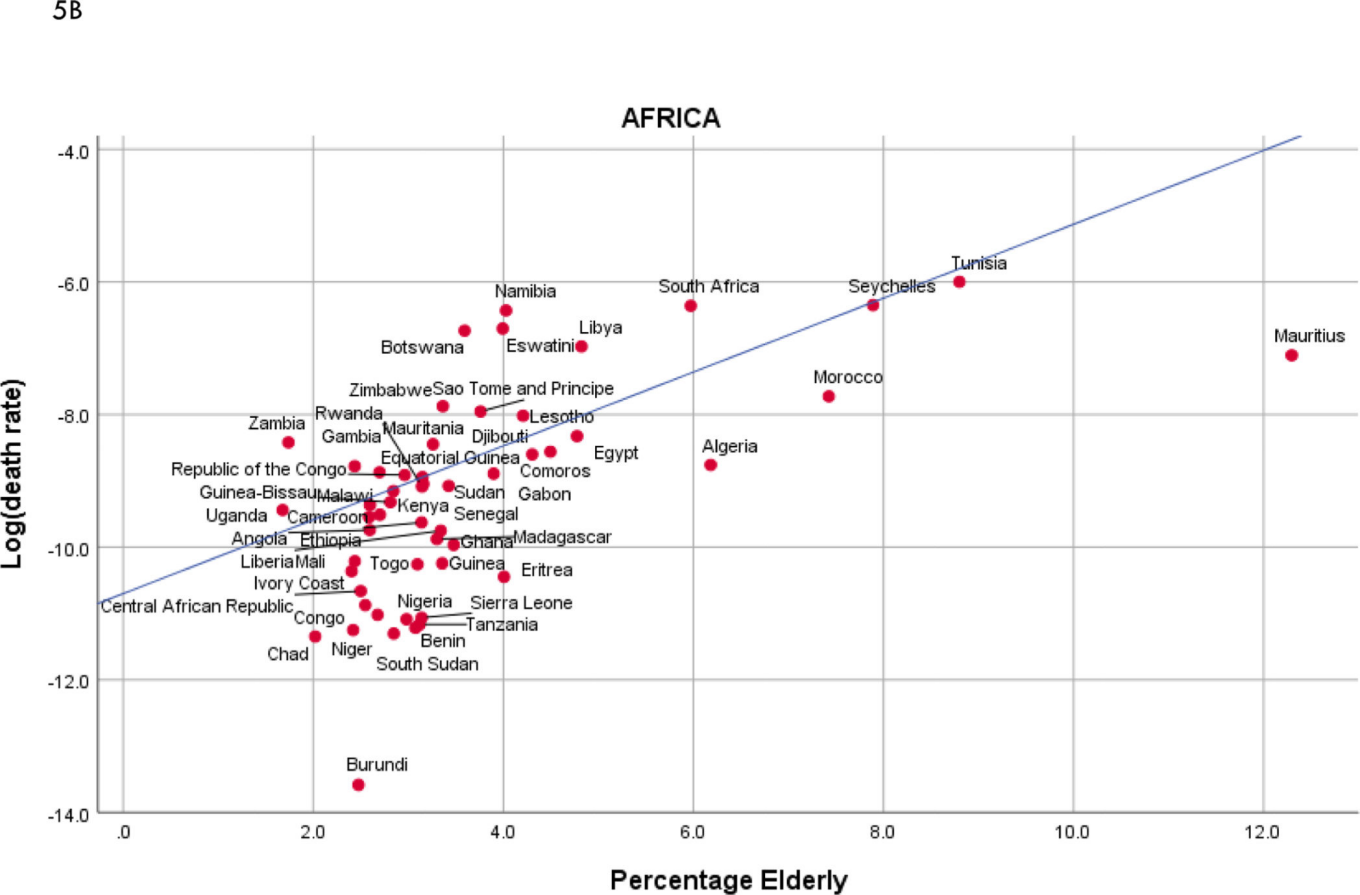

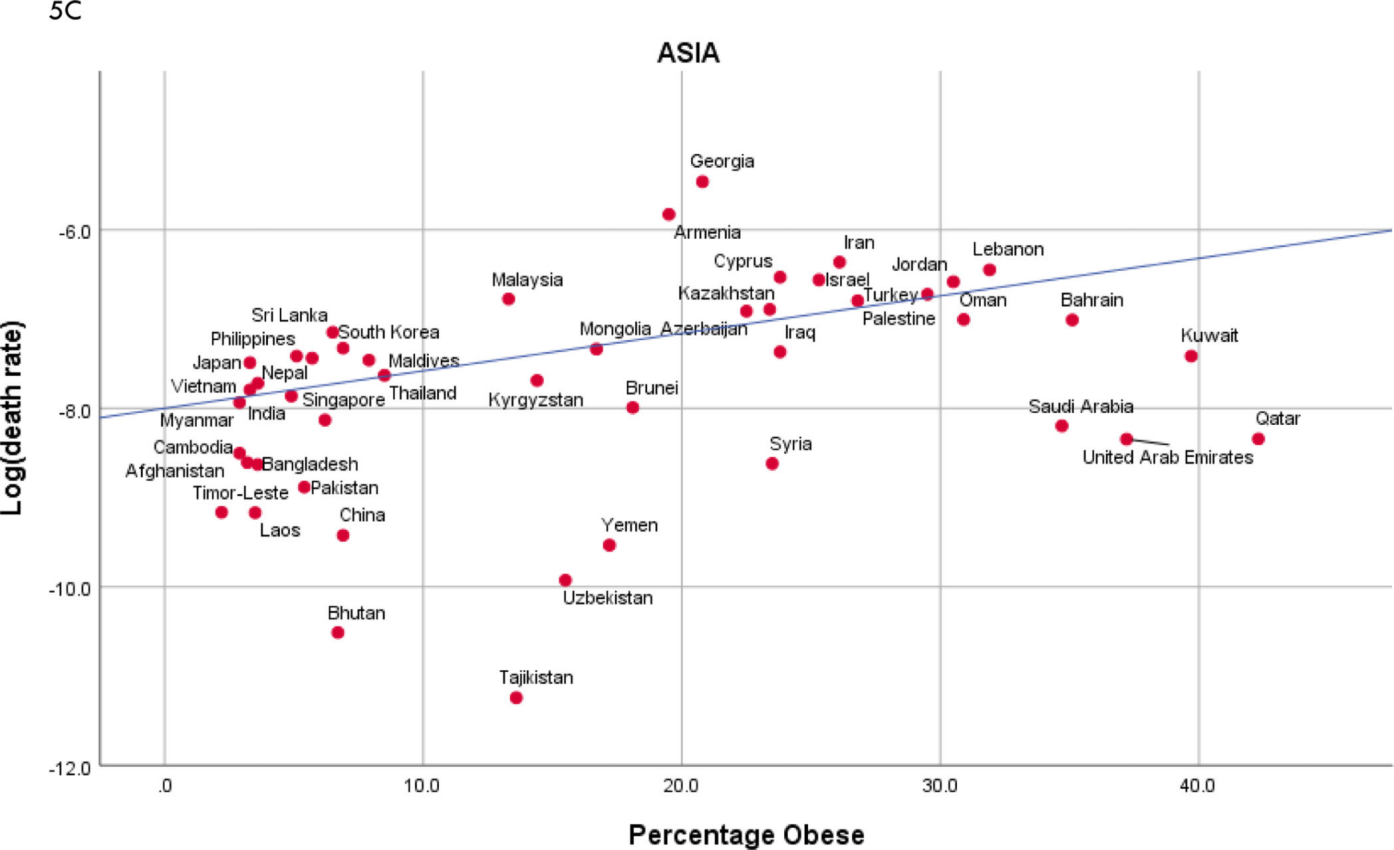

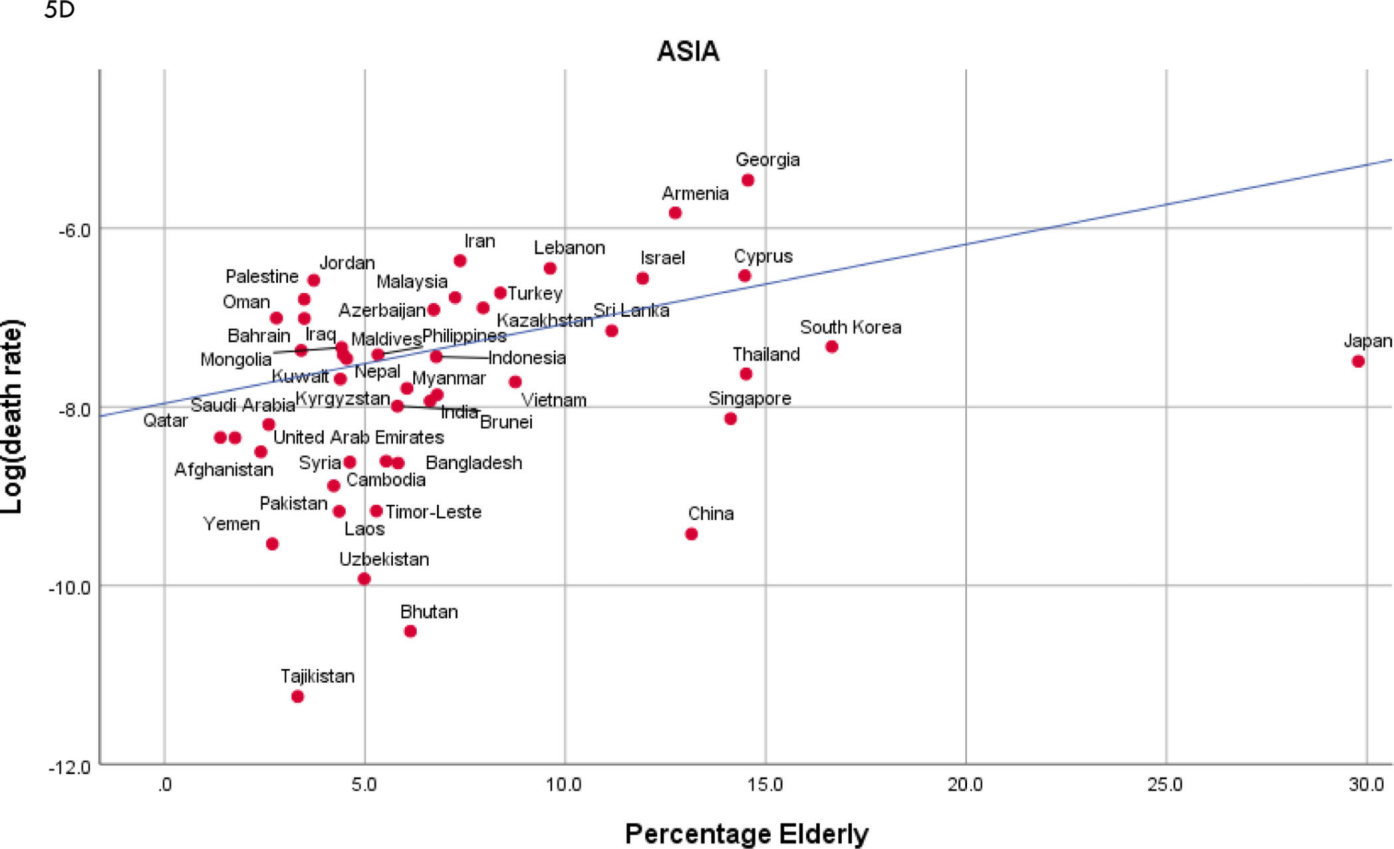

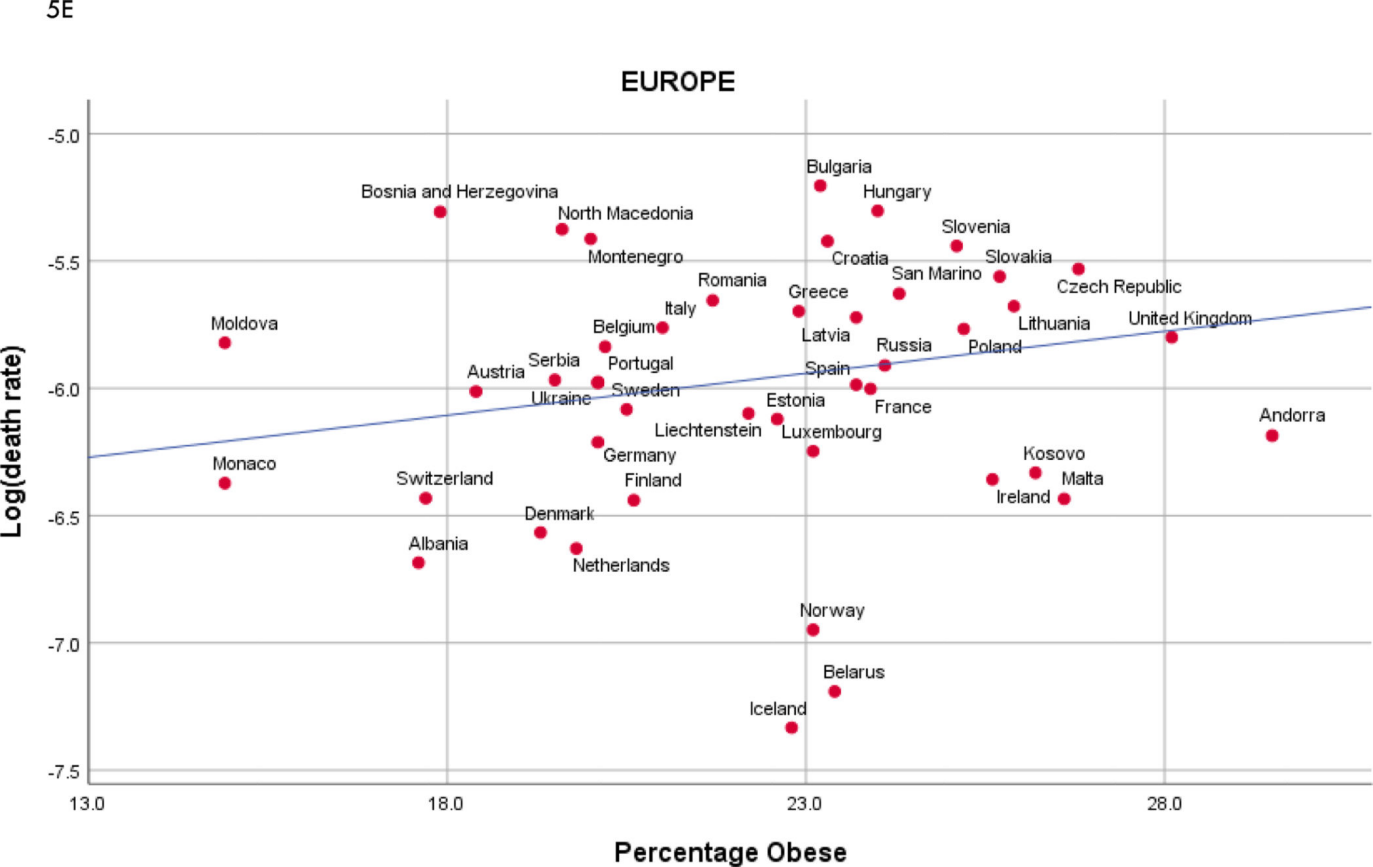

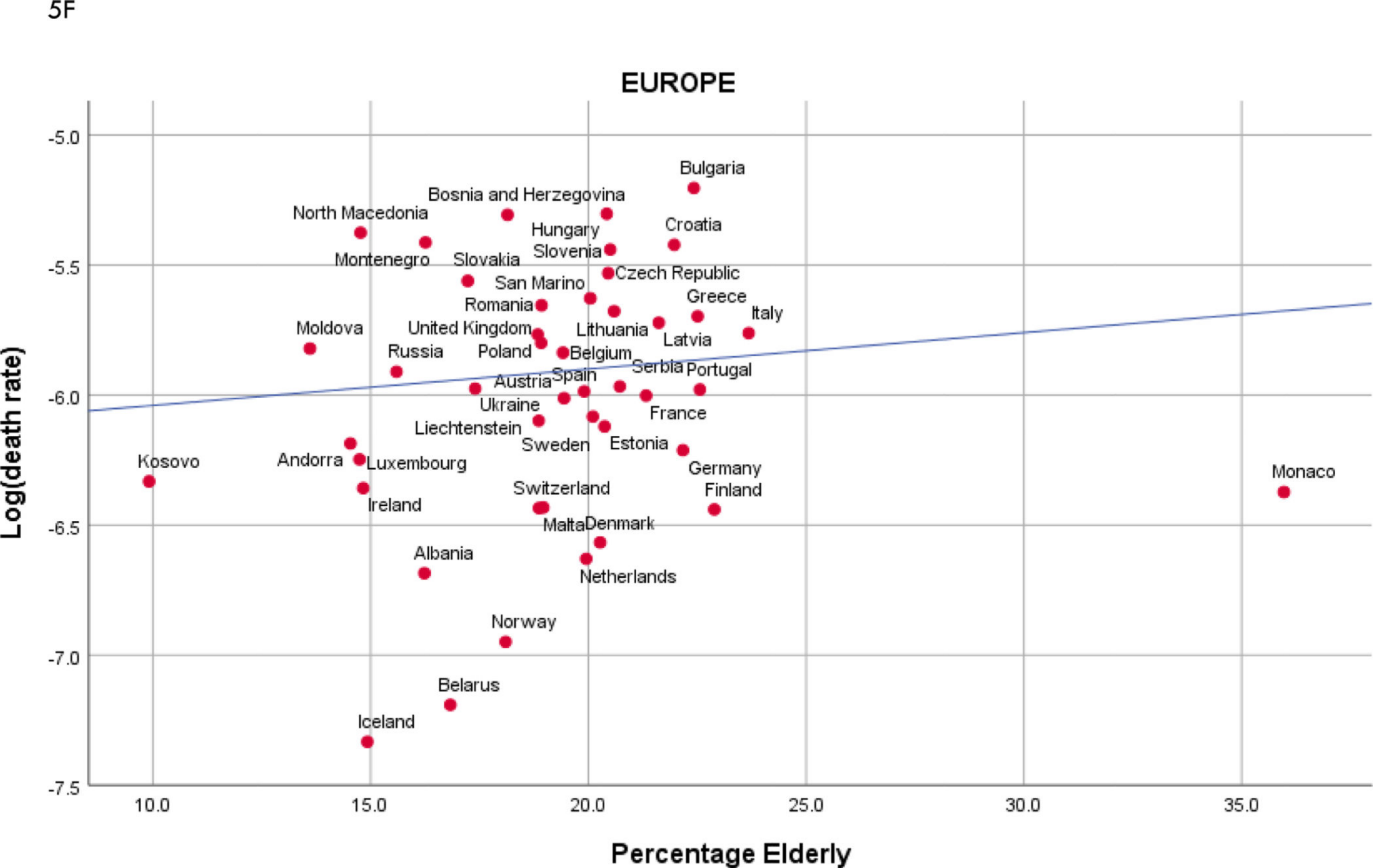

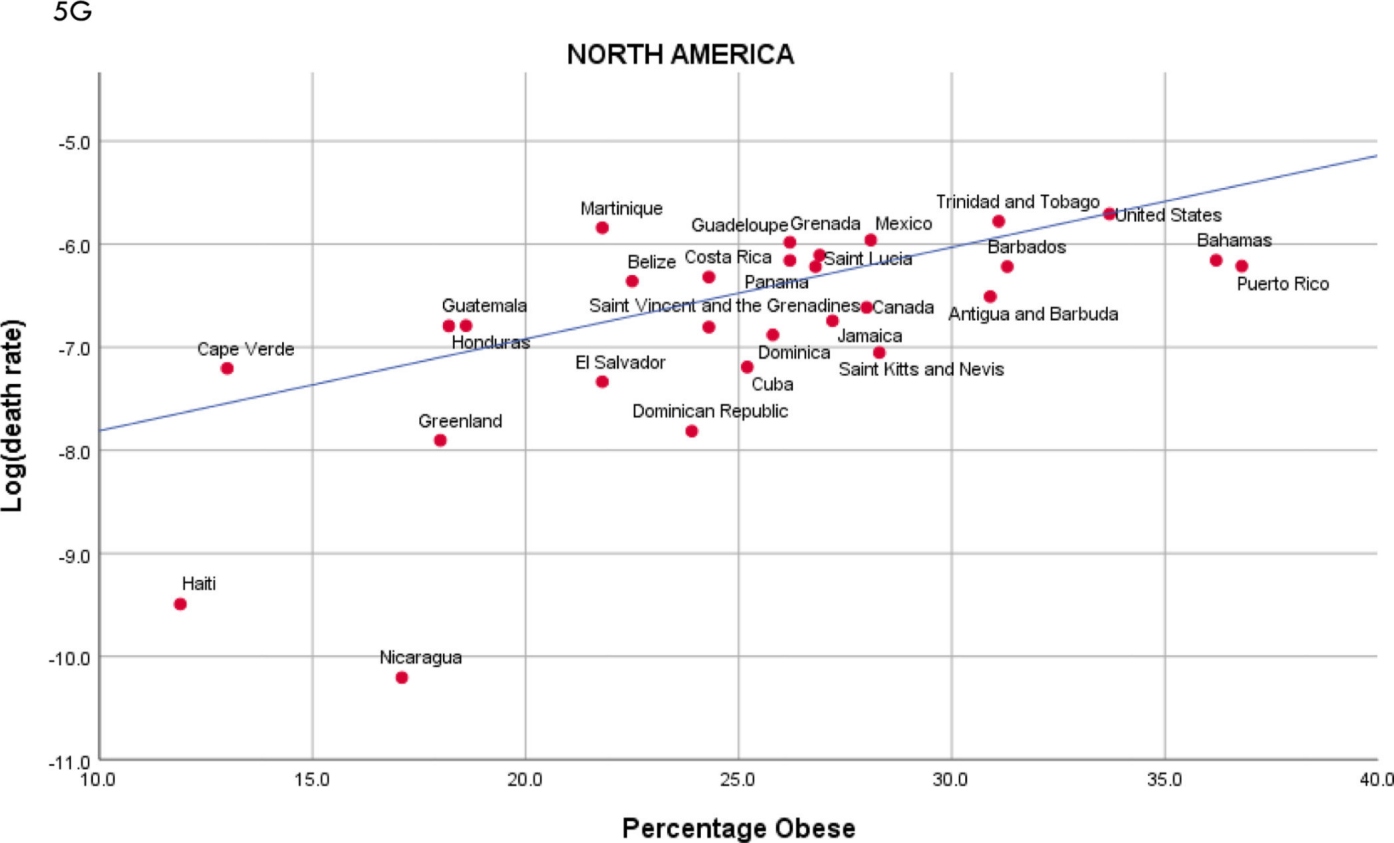

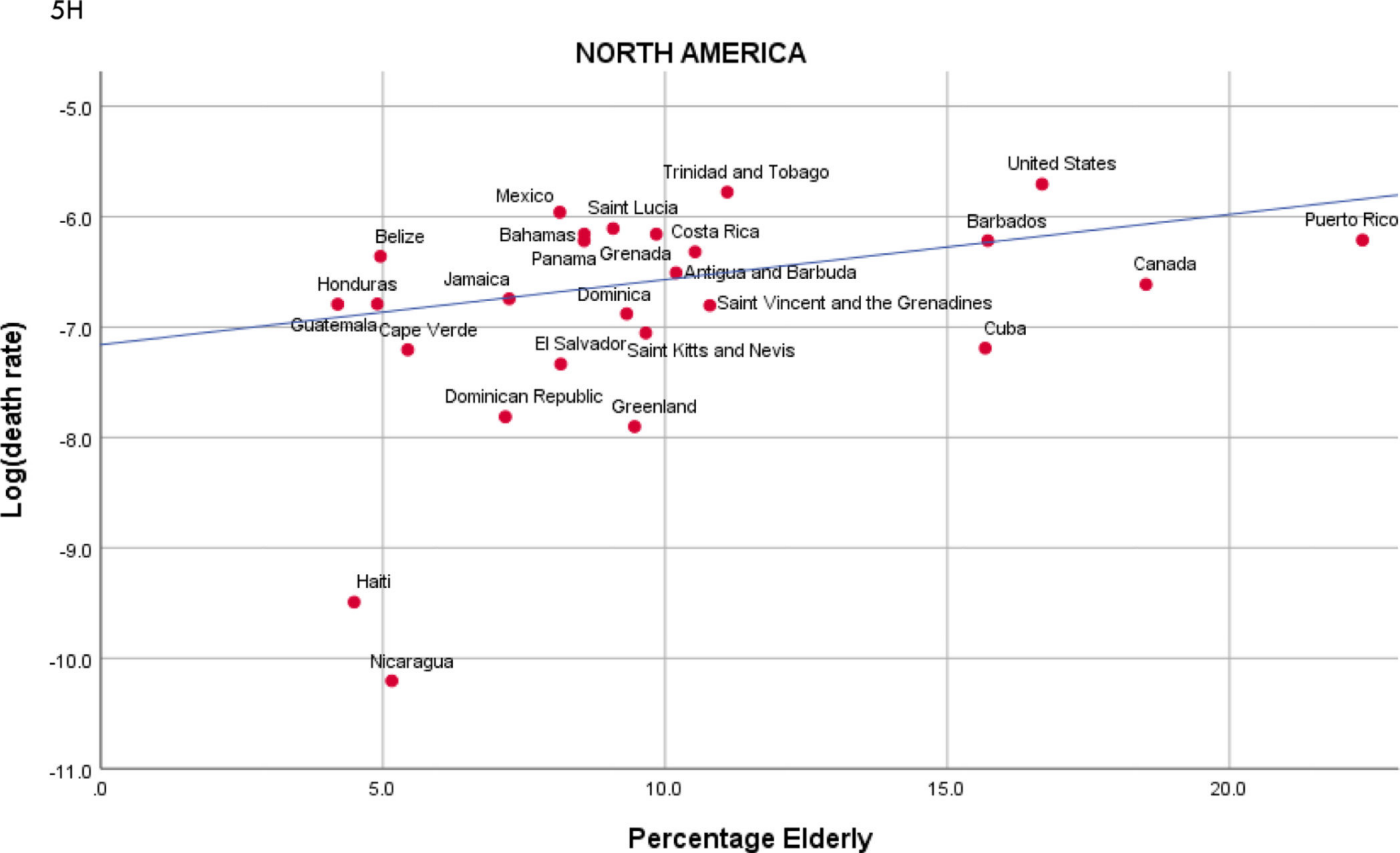

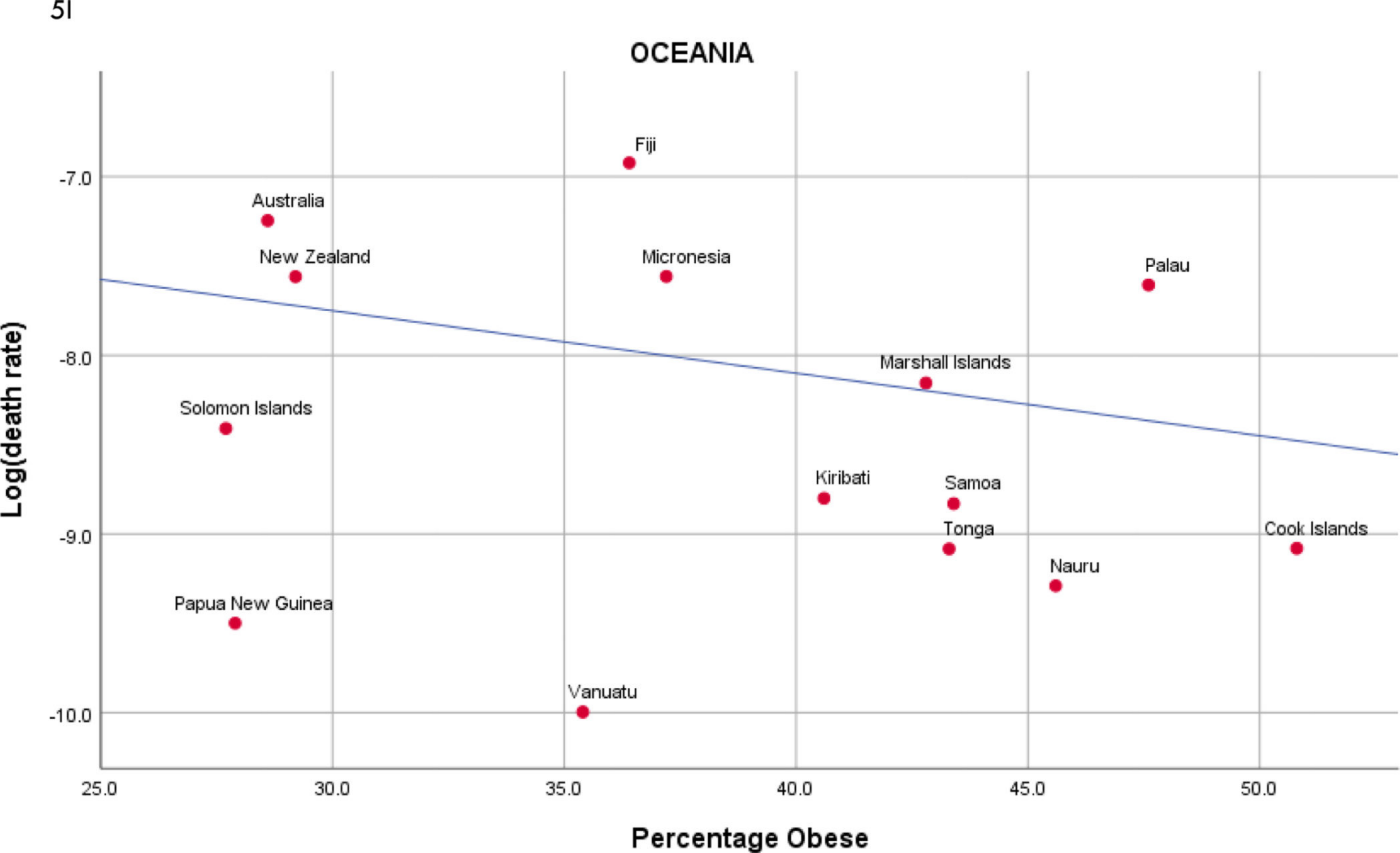

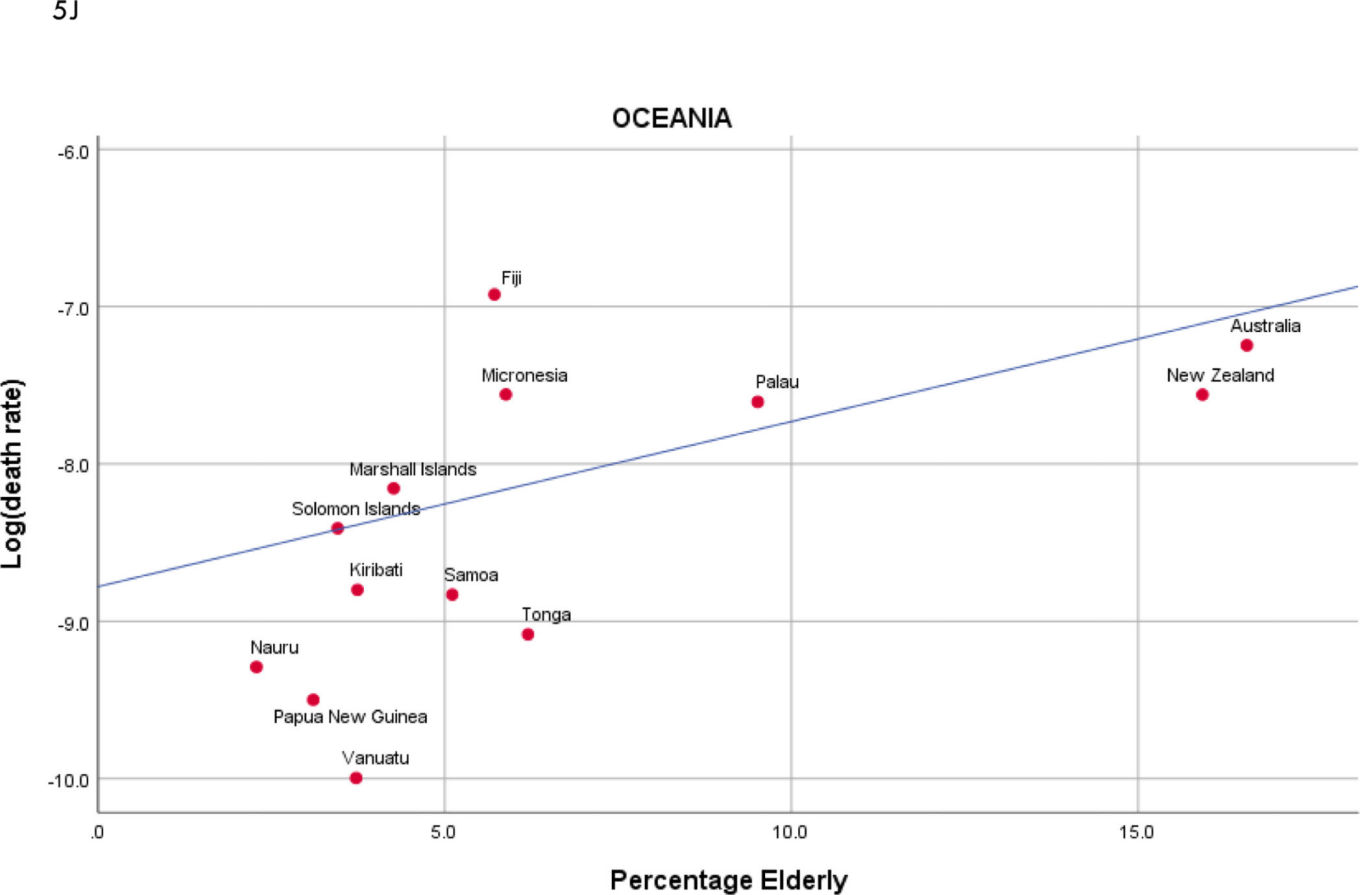

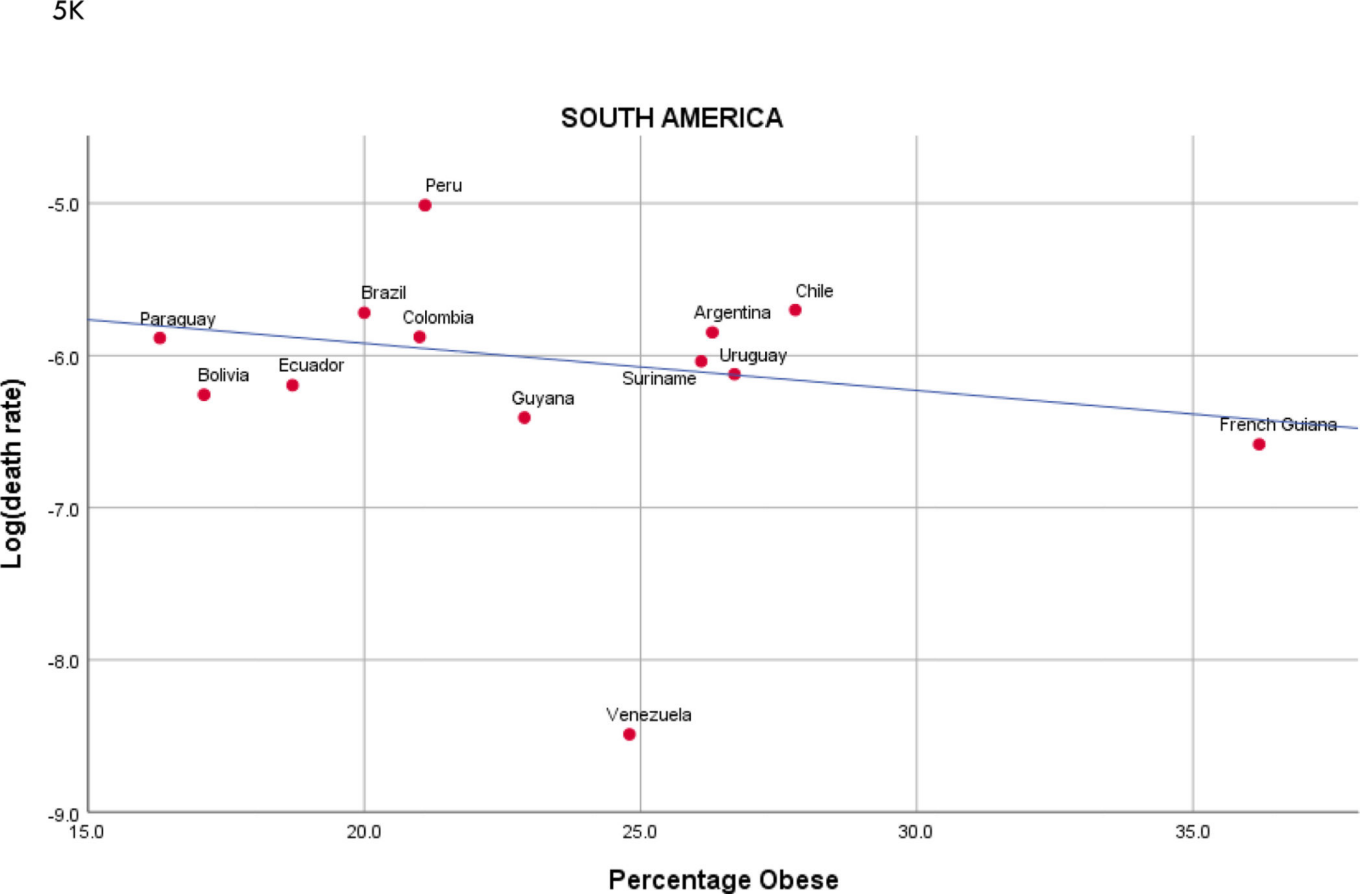

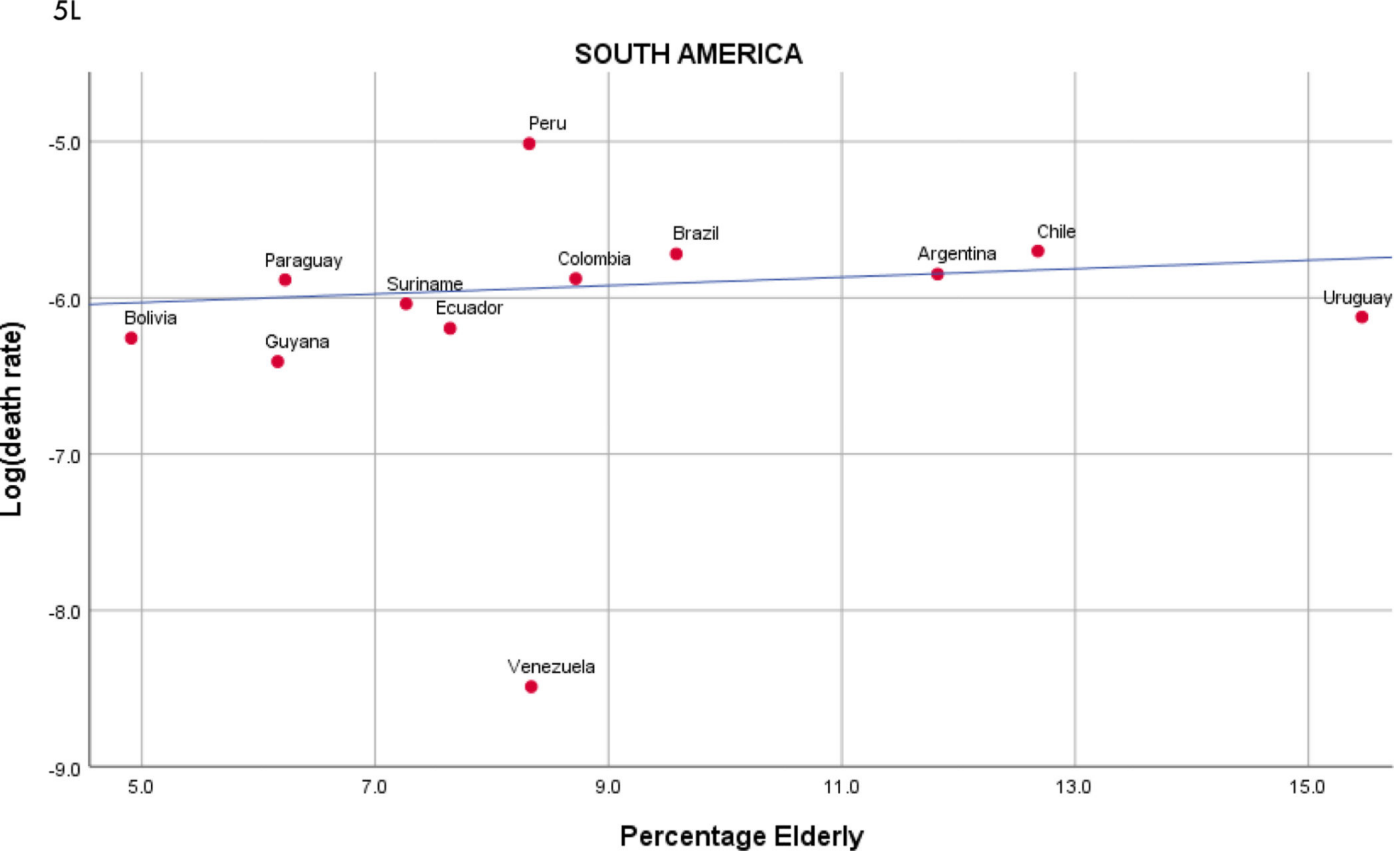
Population percentages of obesity and elderly vs. COVID-19 mortality rates, by region. In each region, annotations of the individual countries are given, as well as the best fitting negative binomial regression line, which is linear on a log scale.

We further elucidate national differences in COVID-19 mortality in Figure 5, where we present graphs of percentage obesity and elderly vs. COVID-19 mortality by region, along with best fitting negative binomial regression lines. Of these graphs, there are 5 plots with significant associations, that is, slopes significantly different from zero: Africa and Asia with both obesity and elderly, and North America with obesity. The regressions in the remaining plots are characterized by slopes not significantly different from zero. Nevertheless, the plots are revelatory concerning country “extremes”, several of which we highlight: Burundi in Africa has little obesity, elderly, or COVID-19 mortality; the proportions of elderly individuals in Japan (Asia) and Monaco (Europe) vastly exceed that of the other countries in their respective regions; Haiti and Nicaragua (North America) and Venezuela (South America) experienced quite low mortality rates in their respective regions; and the mortality rate in Peru (South America) is exceptionally high for the region.

**Figure 6.**
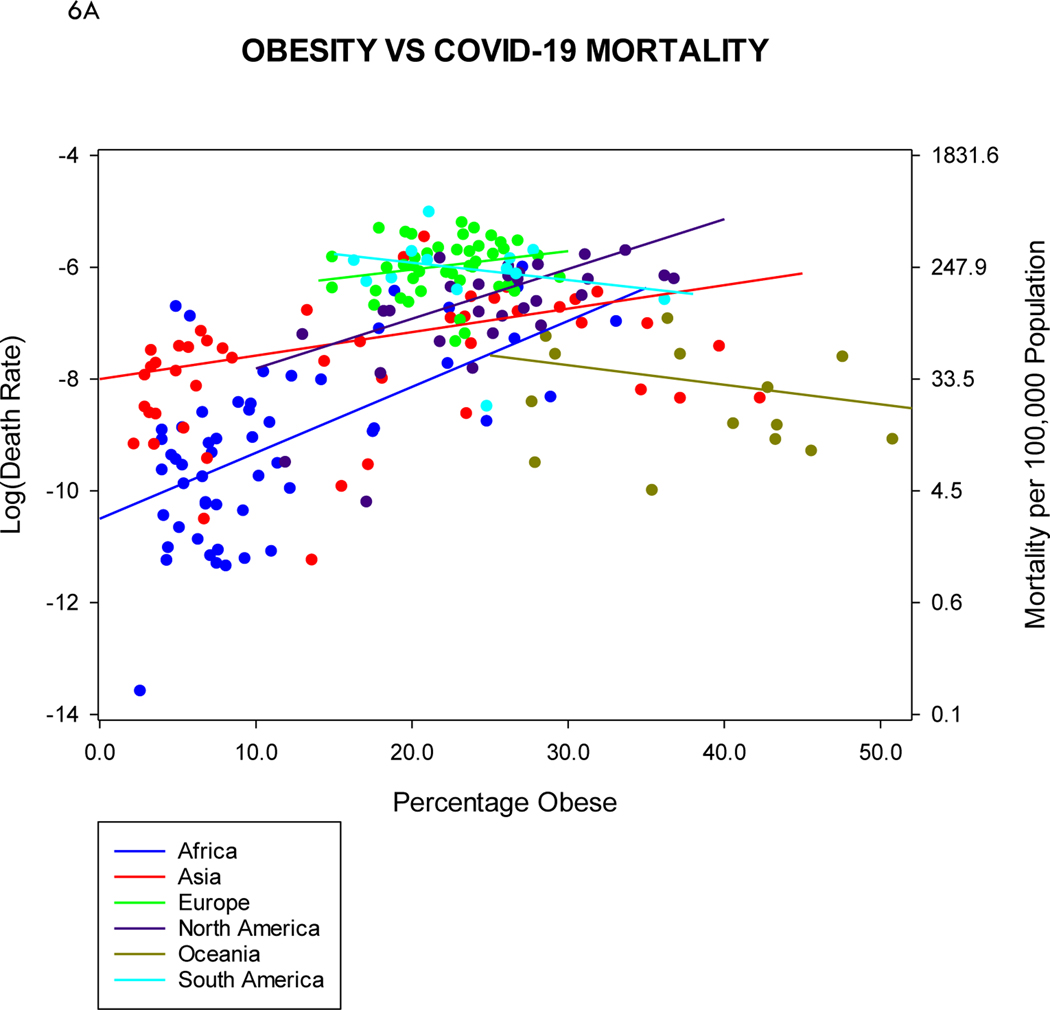

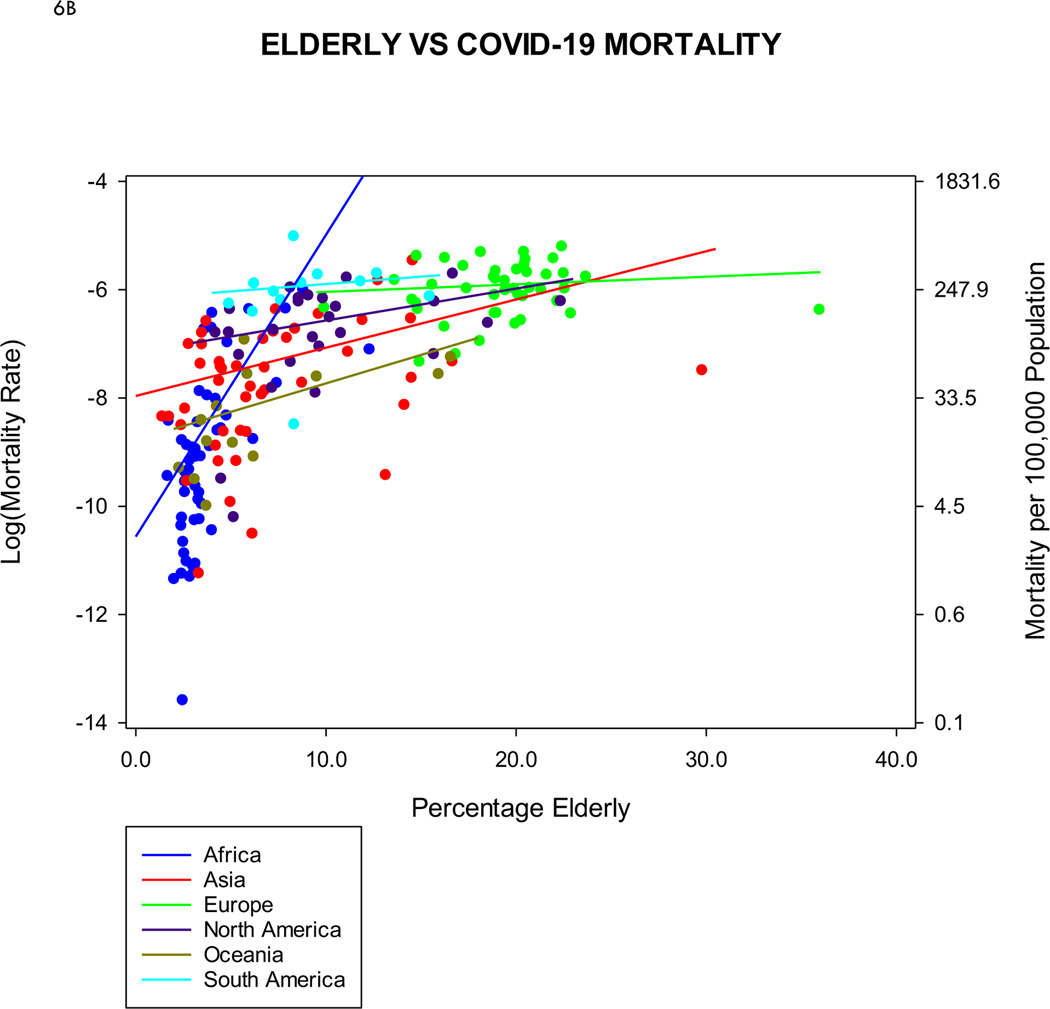
Overall display of percentages of (A) obesity and (B) elderly vs. COVID-19 mortality rates. The regions are color-coded, so that the individual countries as well as the best fitting negative binomial regression lines (both from Figure 5) can be identified. Unlike Figure 5, the scaling along the y-axis (COVID-19 mortality rates) is invariant, so as to afford immediate comparison of countries and regions. As in Figure 5, the left-hand y-axis displays the mortality rates on a log scale. The right-hand y-axis here displays the mortality rates on a conventional scale of deaths per 100,000 population.

In Figure 6, we combine the various regional regressions from Figure 5 into overall displays on the same scale, so as to better illustrate region to region differences. One might declare Africa a decisive winner in the COVID-19 mortality sweepstakes, along with Asia and Oceania (even though obesity is especially pronounced in Oceania).

## Discussion

The COVID-19 pandemic has affected millions of people worldwide. Certain subpopulations, including individuals with obesity and the elderly, appear to be particularly likely to experience severe outcomes and increased mortality rates from COVID-19 infection. That obesity and elderly are considered risk factors for illness and mortality from COVID-19 infection is reasonable from a physiological perspective. Obesity can impair lung function, leading to reduced respiratory capacity and an increased susceptibility to airborne respiratory infections as with COVID-19. In addition, excessive adipose tissue can release inflammatory cytokines that may exacerbate the inflammatory response to viral infections. Similarly, aging is associated with physiological changes to the immune system, including reduced or impaired immune responses, which in turn leads to increasing vulnerability to infections. Obesity may also be a marker of comorbidities such as cardiovascular disease, hypertension, and diabetes, the prevalence of which tends to increase with age. In short, obese and elderly subpopulations are likely to be susceptible to adverse clinical outcomes from COVID-19 infection due to the effects of obesity-related metabolic dysregulation, age-related immune decline, and combined effects of comorbidities.

We therefore postulated that increasing prevalence of obese or elderly subpopulations in countries and territories should be associated with increasing mortality rates from COVID-19. We found wide country-to-country variations in prevalence indices and mortality rates, hence we focused on regional differences, the regions being Africa, Asia, Europe, North America, Oceania, and South America.

Overall, Africa has lower prevalences of obesity and elderly than the other regions. But the prevalences are not homogeneous across the continent: the countries in northern and southern Africa have higher prevalences than the remainder of the continent. This is mirrored in COVID-19 mortality rates, and the significant increase in mortality with increasing prevalence of obesity or elderly is driven by this fact. The markedly low rates of COVID-19 mortality in the interior may well reflect low prevalences of obesity and elderly, but there may be other contributing factors. Preexisting immunity to other diseases such as malaria might confer some protection against COVID-19. Environmental factors might also play a role: the hot climate in Africa may be less conducive to the spread of an airborne virus than colder and drier environments. Interestingly, the uptake of COVID-19 vaccines in Africa has been relatively low, due to such factors as limited vaccine access and delivery infrastructure or vaccine hesitancy. On the other hand, it is possible that COVID-19 mortality in Africa has been underestimated, due to limited testing and reporting capabilities, especially in areas with relatively weak health care systems. Also, racial or ethnic differences between northern Africa and the remainder of the continent may play a contributory role in the observed differences in the obesity and mortality metrics.

In contrast to the north-south geographic demarcation in Africa relative to obesity, elderly, and COVID-19 mortality, the geographic demarcation in Asia is an east-west differential: obesity and COVID-19 mortality are more prevalent in the west, whereas the eastern Asian countries have higher prevalences of the elderly (especially, Japan) and lower prevalences of obesity (perhaps in part a racial or ethnic divide). The countries of eastern Asia tend to have strong and robust healthcare systems with near-universal coverage and timely responses to health care emergencies. Governments imposed strict public health measures, such as widespread testing and contact tracing, and masking, social distancing and quarantine protocols. Also important are cultural norms relating to collective social responsibility leading to widespread acceptance and adherence to governmental public health directives.

Along with the United States, Europe might be considered the epicenter of the COVID-19 pandemic. Mortality rates are quite high across the continent, and the risk factors of obesity and an elderly population are prevalent. Population density might be another factor contributing to rapid virus spread. On the other hand, one might expect that the rapid individual government responses to the pandemic, and the modern healthcare systems available to the populace, would have mitigated the effects of the pandemic.

Haiti and Nicaragua might be considered outliers among the countries of North America given their impressively low mortality rates from COVID-19. Their low proportions of obesity and elderly are important mitigating factors, though these proportions are not exceptional in the region. Here, a comparison of Canada with the United States comparison is instructive: obesity is less prevalent in Canada than the United States, counterbalanced by a slightly greater percentage of the elderly. Canada’s lower COVID-19 mortality rate than the US might well be due to other key factors: a more coordinated and consistent public health response to the pandemic together with greater compliance to public health directives; a government funded healthcare system that provides near universal coverage to the populace; and, lower population density.

Oceania has a high prevalence of obesity, but relatively low percentages of the elderly, and low COVID-19 mortality. The geographic isolation of the Pacific island nations, as well as strict travel restrictions imposed in Australia and New Zealand early in the pandemic, effectively tamped down COVID-19 exposure and ultimately mortality. In addition, there may well be genetic factors contributing to the high obesity rates observed in the island nations.

The countries of South America are relatively homogeneous relative to obesity and elderly profiles, yet exhibit extreme contrasts in COVID-19 mortality. On the continent, Peru has experienced the largest COVID-19 mortality rate, and Venezuela the smallest. In this regard, note that obesity is more prevalent in Venezuela than Peru, and the elderly proportions are relatively similar. One might attribute high COVID-19 mortality to inadequate health care, but it is difficult to ascribe this circumstance solely to Peru. Perhaps high population density has played a contributory role in the spread of the virus in the urban centers of Peru and other South American cities.

It is clear from Figure 6 that overall, COVID-19 mortality rates do tend to increase with increasing prevalence of obesity or elderly populations, on a country basis. Although one might interpret this as affirmation of the orthodoxy, this overall view masks or obscures interregional differences in both risk factors and mortality, as well as intraregional differences (Figure 5). Do the exceptions prove the rule? The most glaring exception is probably Oceania, with high levels of obesity yet low COVID-19 mortality, though the low mortality rate is not altogether surprising. Some extreme country-to-country differences in mortality, e.g., Peru vs. Venezuela, might raise questions: in these cases, we might parse the differences on a finer scale, by delving into regional differences within the individual countries, as we have previously done within the United States^23^. Regardless, there are a handful of countries for which reported COVID-19 mortality rates appear preternaturally low. A pronouncement on a global scale that obesity or elderly status constitute definitive risk factors for COVID-19 mortality overlooks the subtleties engendered by inter- and intraregional differences.

We recognize that our findings are far from conclusive, and are subject to various limitations. With this observational study, we are reporting associations of obesity and elderly with mortality; but stronger associations would perhaps have been obtained if the available mortality data had included detailed demographic information on an individual basis, including co-morbidities. One might also question the accuracy and comprehensiveness of some of the mortality data, especially when arising from countries with weak public health reporting practices and networks. The negative binomial model we have used (or a Poisson model) is entirely appropriate with mortality data, and linearity of rates on a log scale is a commonly accepted metric by public health representatives; nevertheless, our analyses are not at all exhaustive, and monotonicity of rates with quantitative increases of risk factors might be more apparent under suitable transformations or other alternative analytic methodology. On the other hand, we believe our findings are informative and of practical significance, as they provide germane insights into regions’ variabilities in the obesity and elderly characteristics of their populations, and concomitantly in their COVID-19 mortality rates.

## Conclusions.

There are significant differences both between regions and within regions relative to COVID-19 mortality rates, as well as proportions of obese or elderly individuals. A global pronouncement that obesity and elderly constitute definitive risk factors for COVID-19 mortality masks the subtleties engendered by these intra- and interregional differences.

## References

[1] Kang S-J, Jung SI. Age-Related Morbidity and Mortality among Patients with COVID-19. Infect Chemother. 2020;52:154–164. 32537961 10.3947/ic.2020.52.2.154 PMC7335648

[2] Yanez ND, Weiss NS, Romand J-A COVID-19 mortality risk for older men and women. BMC Public Health 2020;20:1742. 33213391 10.1186/s12889-020-09826-8 PMC7675386

[3] COVID-19: review of disparities in risks and outcomes. Public Health England, PHE publications gateway number: GW-1447, 2020.

[4] Dessie ZG, Zewotir T. Mortality-related risk factors of COVID-19: a systematic review and meta-analysis of 42 studies and 423,117 patients. BMC Infect Dis. 2021;21:855. 34418980 10.1186/s12879-021-06536-3 PMC8380115

[5] Foo O, Hiu S, Teare D, A global country-level analysis of the relationship between obesity and COVID-19 cases and mortality. Diabetes Obes Metab 2021;23:2697–2706. 34402152 10.1111/dom.14523 PMC8444639

[6] Gao M, Piernas C, Astbury NM, Associations between body-mass index and COVID-19 severity in 6·9 million people in England: a prospective, community-based, cohort study. Lancet Diabetes Endocrinol 2021;9:350–359. 33932335 10.1016/S2213-8587(21)00089-9 PMC8081400

[7] Poly TN, Islam MM, Yang HC, Obesity and Mortality Among Patients Diagnosed With COVID-19: A Systematic Review and Meta-Analysis. Front Med (Lausanne) 2021;8:620044. 33634150 10.3389/fmed.2021.620044 PMC7901910

[8.] Starke KR, Reissig D, Petereit-Haack G, The isolated effect of age on the risk of COVID-19 severe outcomes: a systematic review with meta-analysis. BMJ Glob Health 2021;6:e006434. 10.1136/bmjgh-2021-006434 PMC8678541 34916273

[9] Dadras O, SeyedAlinaghi SA, Karimi A, COVID‐19 mortality and its predictors in the elderly: A systematic review. Health Sci Rep 2022;5:e657. 35620541 10.1002/hsr2.657 PMC9125886

[10] Paravidino VB, Leite TH, Mediano MFF, Association between obesity and COVID-19 mortality and length of stay in intensive care unit patients in Brazil: a retrospective cohort study. Sci Rep 2022;12:13737. 35962010 10.1038/s41598-022-17197-w PMC9372981

[11] Sawadogo W, Tsegaye M, Gizaw A, Adera T. Overweight and obesity as risk factors for COVID-19-associated hospitalisations and death: systematic review and meta-analysis. BMJ Nutr Prev Health 2022;5:10–18. 10.1136/bmjnph-2021-000375 PMC8783972 35814718

[12] Singh R, Rathore SS, Khan H, Association of Obesity With COVID-19 Severity and Mortality: An Updated Systemic Review, Meta-Analysis, and Meta-Regression. Front Med (Lausanne) 2022; 13: 780872. 10.3389/fendo.2022.780872 PMC9205425 35721716

[13] Habis Y, Alsilmi R, Alirbidi L, Effect of Obesity on Clinical Outcomes in COVID-19 Patients. Cureus 2023;15: e33734. 36793811 10.7759/cureus.33734 PMC9922939

[14] Harris E Most COVID-19 Deaths Worldwide Were Among Older People. JAMA 2023;329:704. 10.1001/jama.2023.1554 36790826

[15] Tadayon NB, Rayner DG, Shokraee K, Obesity as an independent risk factor for COVID-19 severity and mortality. Cochrane Database Syst Rev 2023;5:CD015201. 10.1002/14651858.CD015201 PMC10207996 37222292

[16] Wong MK, Brooks DJ, Ikejezie J, COVID-19 Mortality and Progress Toward Vaccinating Older Adults — World Health Organization, Worldwide, 2020–2022. MMWR Morb Mortal Wkly Rep 2023;72:113–118. 36730046 10.15585/mmwr.mm7205a1 PMC9927068

[17] World Health Organization Coronavirus Dashboard. https://covid19.who.int/ Accessed 17 March 2023.

[18] World Bank DataBank. Population ages 65 and above (% of total population). https://data.worldbank.org/indicator/SP.POP.65UP.TO.ZS Accessed 1 April 2023.

[19] Renew Bariatrics Report: Obesity Rates by Country 2022. https://renewbariatrics.com/obesity-rank-by-countries/ Accessed 17 March 2023.

[20] Gardner W, Mulvey EP, Shaw EC. Regression Analyses of Counts and Rates: Poisson, Overdispersed Poisson, and Negative Binomial Models. Psychological Bulletin 1995;118:392–404. 7501743 10.1037/0033-2909.118.3.392

[21] Cameron AC, Trivedi PK. Regression Analysis of Count Data. Cambridge University Press: 1998.

[22] Hilbe JM. Negative Binomial Regression. Cambridge University Press: 2007.

[23] Koziol JA, Schnitzer JE. State Government Policy Responses to the COVID-19 Pandemic in the United States 2022–2022: Concordant Heterogeneity? Medical Research Archives, v. 11, n. 4, apr. 2023. ISSN 2375–1924. Available at: <https://esmed.org/MRA/mra/article/view/3693>. 10.18103/mra.v11i4.3693 PMC10421646 37575472

